# FGF13 is not secreted from mouse neurons

**DOI:** 10.1172/jci.insight.195998

**Published:** 2025-11-25

**Authors:** Mattia Malvezzi, Haiying Zhang, Patrick Towers, David C. Lyden, Steven O. Marx, Geoffrey S. Pitt

**Affiliations:** 1 Cardiovascular Research Institute, and; 2 Children’s Cancer and Blood Foundation Laboratories, Departments of Pediatrics, and Cell and Developmental Biology, Drukier Institute for Children’s Health, Meyer Cancer Center, Weill Cornell Medicine, New York, New York, USA.; 3 Division of Cardiology, Department of Medicine, and Department of Molecular Pharmacology and Therapeutics, Vagelos College of Physicians and Surgeons, Columbia University, New York, New York, USA.

**Keywords:** Cell biology, Neuroscience, Signal transduction

## Abstract

FGF13, a noncanonical fibroblast growth factor (FGF) and member of the fibroblast growth factor homologous factor (FHF) subset, lacks a signal sequence and was previously reported to remain intracellular, where it regulates voltage-gated sodium channels (VGSCs) at least in part through direct interaction with the cytoplasmic C-terminus of VGSCs. Recent reports suggest FGF13 is secreted and regulates neuronal VGSCs through interactions with extracellular domains of integral plasma membrane proteins, yet supportive data are limited. Using rigorous positive and negative controls, we show that transfected FGF13 is not secreted from cultured cells in a heterologous expression system, nor is endogenous FGF13 secreted from cultured neurons. Furthermore, using multiple unbiased screens including proximity labeling proteomics, our results suggest FGF13 remains within membranes and is unavailable to interact directly with extracellular protein domains.

## Abstract 2

Recent results suggested that FGF13 is secreted from neurons. Here, we show that FGF13 is not secreted from mouse neurons, thus limiting its possible functions.

## Graphical abstract

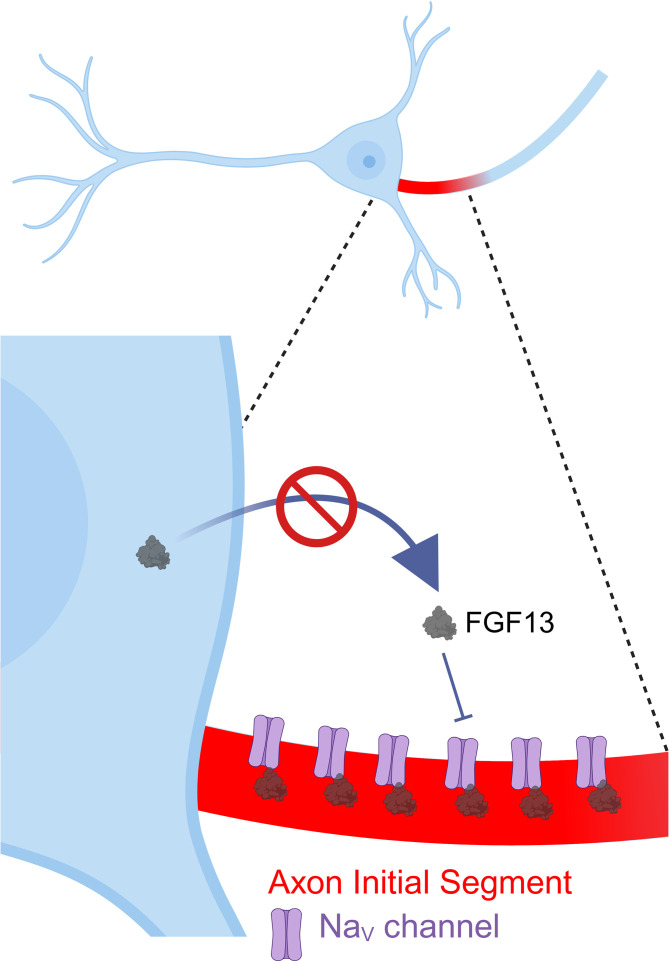

## Introduction

For nearly 30 years since they were first discovered, fibroblast growth factor homologous factors (FHFs) have been described as members of the fibroblast growth factor (FGF) subfamily that differs from canonical FGFs in 2 key aspects: they lack a signal peptide and therefore are not secreted, and they do not bind and activate FGF receptors at physiological concentrations (1). Instead, they have been shown to act intracellularly. There are 4 FHFs—FGF11–14 (FHF3, 1, 2, and 4, respectively)—and although their best-documented role is the regulation of voltage-gated sodium channel (VGSC) activity (2–11), achieved via direct binding to the VGSC C-terminal domain (8, 12–15), data support VGSC independent roles, such as microtubule stabilization (16) and synaptic transmission regulation (17). Furthermore, FHFs, specifically FGF13, are overexpressed in many tumors (18–20), although their role remains unclear.

FHF genes generate multiple isoforms via alternative splicing (21). Among FGF13 isoforms, the brain expresses predominantly FGF13S (aka, FGF13A or FHF2A) and FGF13VY, with FGF13S expressed mainly in excitatory neurons (22, 23). FGF13S is highly concentrated at the neuron axon initial segment (AIS), where it regulates VGSC current density (10).

Several recent studies challenged the paradigm that FHFs are not secreted and do not activate FGF receptors (24–27). Specifically, FGF13S was suggested to be secreted from neurons, causing inhibition of VGSCs and a reduction in the intrinsic excitability at the AIS via binding to the extracellular receptor LRRC37B, with implications for epilepsy and neurodevelopmental disorders (27). FGF13S secretion, however, was analyzed only in a heterologous system and without the benefit of a complement of positive and negative controls. Whether FGF13S is secreted from neurons and has a consequent extracellular physiological role is not known, thus clouding an understanding of its physiological functions.

Here we show that FGF13S is not secreted from HEK293 cells, even in the presence of stress, nor from isolated mouse hippocampal neurons, while known secreted proteins were readily found in the extracellular space. Any FGF13S detected within the extracellular medium of cultured HEK293 cells was restricted to large extracellular vesicles (LEVs) and, to a lesser extent, to small extracellular vesicles (SEVs), which include exosomes. In an orthogonal analysis, we performed in vivo proximity labeling proteomics in the mouse brain and found that FGF13S near neighbors are cytoplasmic, nonsecreted proteins. These results provide clarification that FGF13 remains cytoplasmic and does not function as secreted protein.

## Results

Assays for determining whether a transfected protein is secreted from cultured cells and available to bind to extracellular receptors generally rely on detecting the protein in the extracellular medium of the transfected cells, after clearing the medium of dead or dying cells, and of extracellular vesicles in which the queried protein may be present yet unavailable to interact directly with extracellular receptors. To test if FGF13S is secreted from HEK293 cells (in which FGF13S is not endogenously expressed), we transiently transfected *FGF13S* and then used a multistep differential centrifugation protocol designed to minimize contamination from FGF13S released from dead or dying cells or FGF13S within extracellular vesicles. We also transfected HA-tagged adipsin, an adipokine secreted from adipose tissue (28), as a positive control for secretion. Cells were cultured for 24 hours after transfection with either *FGF13S* or adipsin and then incubated for 24 hours in fresh, serum-free medium. Serum was removed for 2 reasons: (a) to allow us to concentrate the cleared extracellular medium and load comparable amounts of protein among the different fractions, which is not possible when serum is present because of the large amount of albumin, that creates artifacts when running the samples on a polyacrylamide gel, even when the samples are not concentrated (Supplemental Figure 1A; supplemental material available online with this article; https://doi.org/10.1172/jci.insight.195998DS1); and (b) to reproduce the experimental conditions in previous studies in which FGF13S was reportedly detected in the serum-deprived extracellular medium of cultured cells (25).

**Figure 1:**
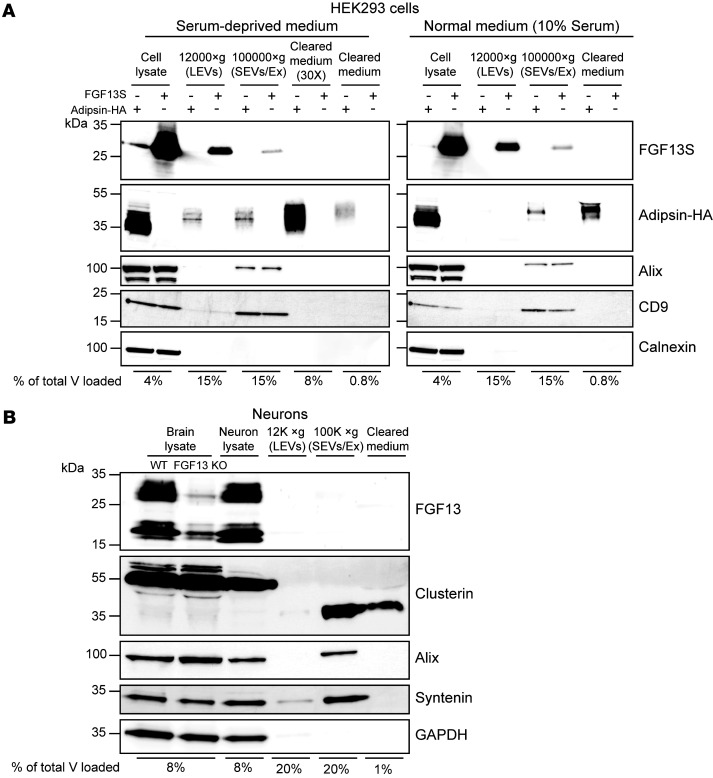
FGF13S is not detected in cleared extracellular medium of HEK293 cells or cultured mouse neurons. (**A**) Western blot of cell lysates, extracellular vesicles, and cleared medium derived from HEK293 transiently transfected with *FGF13S* or the secreted protein HA-tagged adipsin (adipsin-HA) and subjected to multistep centrifugation protocol in serum-deprived conditions (left) or in normal culture conditions (10% serum; right). Alix (96 kDa) and CD9 (24 kDa) were used as SEV or exosome markers; calnexin (90 kDa) was used as an intracellular marker. Secreted adipsin-HA had a higher molecular weight as a consequence of post-translational modifications (28). Cell lysate (8 μg) was loaded. (**B**) Western blot of cell lysates, extracellular vesicles, and cleared medium from cultured mouse hippocampal neurons after 12 DIV in culture. Whole-brain lysates from WT and FGF13 brain KO were used to validate the detection of FGF13 isoforms. Clusterin was used as a positive control for secretion in neurons (nonsecreted clusterin precursor: 55 kDa; cleaved, secreted clusterin: 35 kDa). Alix (96 kDa) and syntenin (34 kDa) were used as SEV and exosome markers; GAPDH (36 kDa) was used as an intracellular marker. Brain or neuronal lysate (15 μg) was loaded. The percentage of total volume (V) loaded indicates the fraction of the sample loaded on each gel relative to the total volume.

Extracellular medium was collected and subjected to sequential centrifugations. A first, low-speed (4,000*g*) centrifugation cleared the medium of any cell debris. The pellet was discarded, and the cleared medium was then centrifuged at 12,000*g* to precipitate LEVs and then at 100,000*g* to collect SEVs, including exosomes. LEVs and SEVs were resuspended, lysed, and loaded on gels for Western blot analysis together with the concentrated (30×) cleared medium, in which true secreted proteins are expected. Indeed, abundant adipsin signal was detected in the cleared medium, but FGF13S was not (Figure 1A). It is worth noting that not only did we not detect adipsin in the extracellular medium, we found it had a higher molecular weight (~40–50 kDa) than the adipsin detected in the cell lysate, reflecting the processing (glycosylation) of the intracellular, presecreted 28 kDa protein (28), (the apparent molecular weight of the protein is larger than native adipsin due to the presence of the HA tag), providing validation for our medium-clearing protocol. The broad band spanning approximately 10 kDa observed in the cleared medium likely reflects variable glycosylation states (28).

In addition to detecting FGF13S in the cell lysate, we detected FGF13S in LEVs and, to a lesser extent, in SEVs, whereas adipsin was barely detected in either fraction. We further confirmed the identity of the extracellular vesicle (EV) fractions with validated markers. Specifically, Alix and CD9, 2 well-established exosomal markers (29), were found in the SEVs/exosome fraction but not in LEVs or in the cleared medium (Figure 1A). Calnexin, an ER membrane protein, was observed in the cell lysate but not found in any fraction (Figure 1A), confirming the successful removal of cell debris or cytoplasmic-only proteins from the EVs and medium. Thus, our clearing protocol enriches for LEVs, SEVs, and cleared extracellular medium while eliminating cytoplasmic debris from dead or dying cells. Taken together, these results show FGF13S is not secreted from HEK293 cells but it is found in EV vesicles, possibly explaining its detection in previous secretion studies that did not use sequential centrifugation to separate EVs from extracellular material (25, 27).

Because these experiments were performed under conditions of serum deprivation for the technical reasons we have noted, the cells were under stress conditions. Although this strategy was integral to previous studies in which FGF13S was detected in the extracellular medium (25), we asked whether stress was required for HEK293 cells to release FGF13S in EVs by also performing the sequential centrifugation protocol on cells for which the 10% serum was not removed. Although the amount of cleared medium able to be loaded on the gel was 10-fold less due to the presence of serum components, primarily large amounts of albumin, that interfere with subsequent Western blotting, we still clearly detected adipsin in the extracellular medium (Figure 1A, right). For direct comparison between the serum-free and the serum-maintained conditions, we loaded the same reduced fraction (0.8%) of extracellular material and observed secreted adipsin for both conditions (Figure 1A, left). Importantly, detection of FGF13S in both LEVs and SEVs was unaffected by the presence or absence of serum, as was the detection of the positive and negative markers for EVs (Figure 1A). Taken together, these results show FGF13S can be detected in EVs of HEK239 cells independent of stress, and FGF13 is not detected in the cleared extracellular medium. Thus, we conclude FGF13S is not secreted from HEK293 cells.

The proposed physiological role for secreted FGF13S is to regulate VGSCs in the brain, so we specifically assessed whether FGF13 is secreted from neurons. Hippocampal neurons from WT mouse brain (P0) were cultured for 12 days (12 DIV), allowing neuronal maturation and testing for secretion. After 12 DIV, the medium was collected, cell debris was removed via centrifugation at 4,000*g*, and the extracellular medium was cleared via the same sequential spin protocol used for HEK293 cells to separate LEVs (12,000*g*) and SEVs (100,000*g*). Cleared extracellular medium was not concentrated, due to the high amounts of supplements in the medium (albumin and hormones) necessary to culture neurons, but which generate gel artifacts (Supplemental Figure 1B). Cell lysates and the fractions obtained from the sequential spins were analyzed by Western blots. Whole-brain lysates from WT or neuronal-specific *Fgf13* knockout (*Nes-cre;Fgf13^fl/Y^*) mice provided validation of the antibody for detection of FGF13 brain isoforms, including FGF13S. GAPDH, a cytoplasmic protein, was not detected in the extracellular medium or in EVs (Figure 1B), confirming successful removal of cell debris. Alix and syntenin, 2 exosomal markers (29), were readily detected in the SEVs fraction but not in the cleared medium, showing successful separation of EVs. Similar to the HEK293 cell experiments, the lack of detection of the cytoplasmic marker and the exosomal markers in the cleared medium demonstrates that the cleared medium is free from cellular debris and EVs. In contrast, clusterin, a disulfide-linked heterodimeric protein conventionally secreted from neurons (30, 31) and that undergoes cleavage and glycosylation of its 55 kDa precursor (32), was detected in the cleared extracellular medium (Figure 1B). Consistent with previous reports (33, 34), we also detected clusterin in the SEV fraction. We additionally established that clusterin was secreted and accumulated in the extracellular medium, because removing the medium after 11 DIV and replacing it with fresh medium for 1 additional day before collection yielded too little clusterin accumulation for detection (Supplemental Figure 2). Under these conditions, we did not detect FGF13S (or any other known FGF13 splice variant) in the medium, even though multiple FGF13 isoforms are present in neuronal lysates (Figure 1B). In contrast to the HEK293 experiments, we did not detect any FGF13 in the EV fractions, suggesting that its presence in EVs might be cell-type or tissue specific or dependent on stimuli such as stress. Together, these results do not support the hypothesis FGF13S is secreted from neurons or that FGF13S is released into the extracellular space from exosomes.

To provide an alternative means to assess if FGF13S is secreted from neurons, we performed in vivo proximity labeling proteomic profiling in mouse brain. We reasoned that if FGF13S is secreted, either through the conventional pathway or via a nonconventional pathway, it should be in the vicinity of proteins involved in these processes or other secreted proteins, and that at least a subset of those proteins would be detected by proximity labeling. Indeed, in vivo proximity labeling proteomic profiling has been used successfully to characterize secreted or extracellular proteins in various tissues and organs, including the brain (35, 36). Therefore, we generated transgenic mice with a cDNA encoding the biotin ligase TurboID (37) and an HA tag fused to the N-terminus of FGF13S under the control of a tetracycline-inducible promoter. We note that placing TurboID at the N-terminus could prevent FGF13S secretion by blocking a signal sequence, yet a distinguishing feature of FGF13S (like other members of the FHF subfamily) is the absence of a signal sequence (38). These mice were crossed with mice carrying a brain-specific (neurofilament heavy polypeptide, *Nefh*, promoter), tetracycline-controlled transactivator protein (tTA) transgene to achieve expression in the brain.

**Figure 2:**
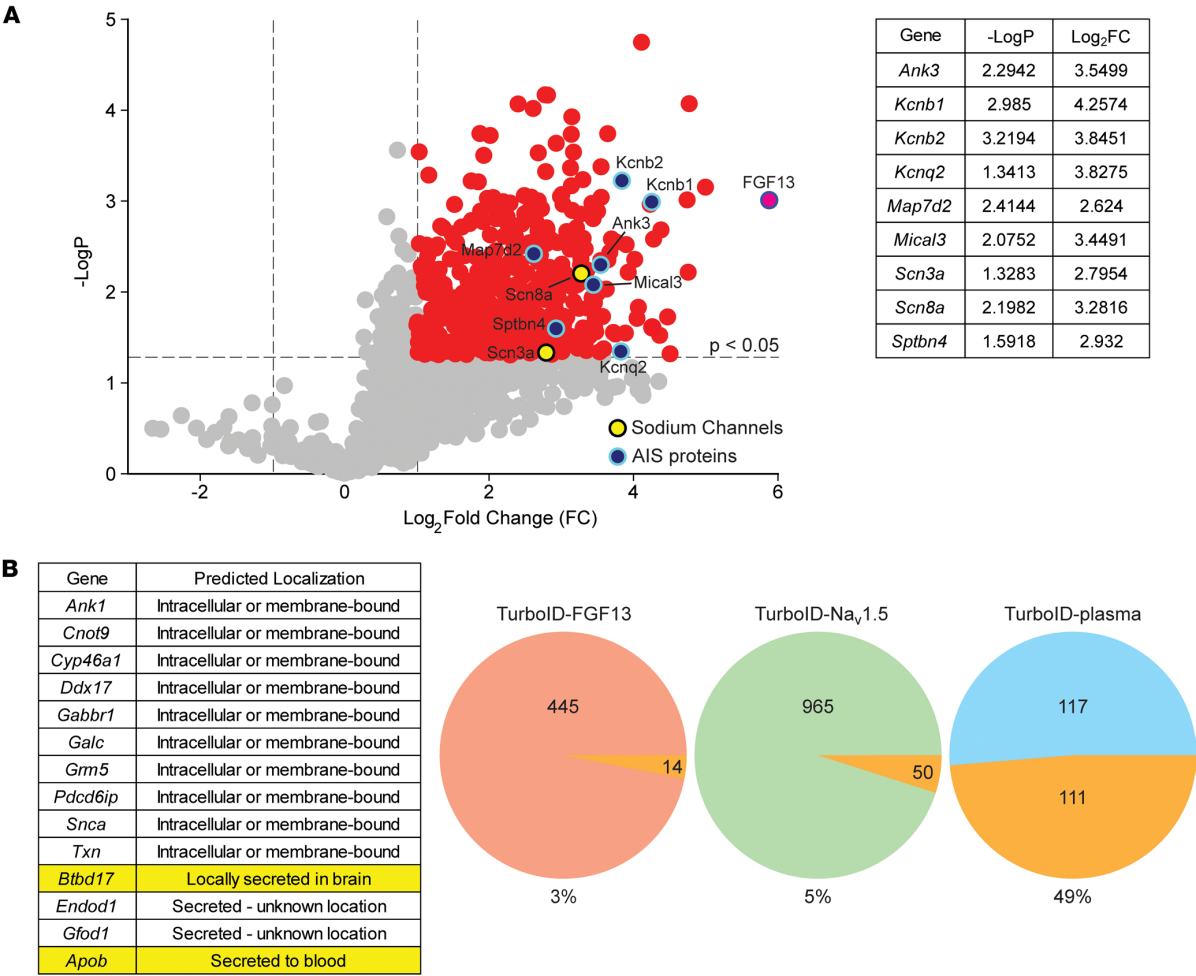
In vivo brain TurboID proximity proteomics shows FGF13S is not in the vicinity of secreted proteins or in proteins involved in secretion processes. (**A**) Volcano plot of proteins enriched in biotin-injected animals compared with control animals. Red indicates highly enriched proteins, with a fold-change >2; pink indicates FGF13; yellow indicates sodium channels; and blue indicates axon initial segment proteins. Statistics for these proteins are shown in the accompanying table. (**B**) Pie charts showing the overlap between the human secretome database of secreted protein (41) and our TurboID-FGF13S (red) dataset, the TurboID-Na_v_1.5 dataset (42) (green), and a dataset of secreted protein detected by TurboID proximity proteomics (cyan) (36). The full list of overlapping proteins found in our study, with their predicted localization, is shown in the accompanying table.

**Figure 3:**
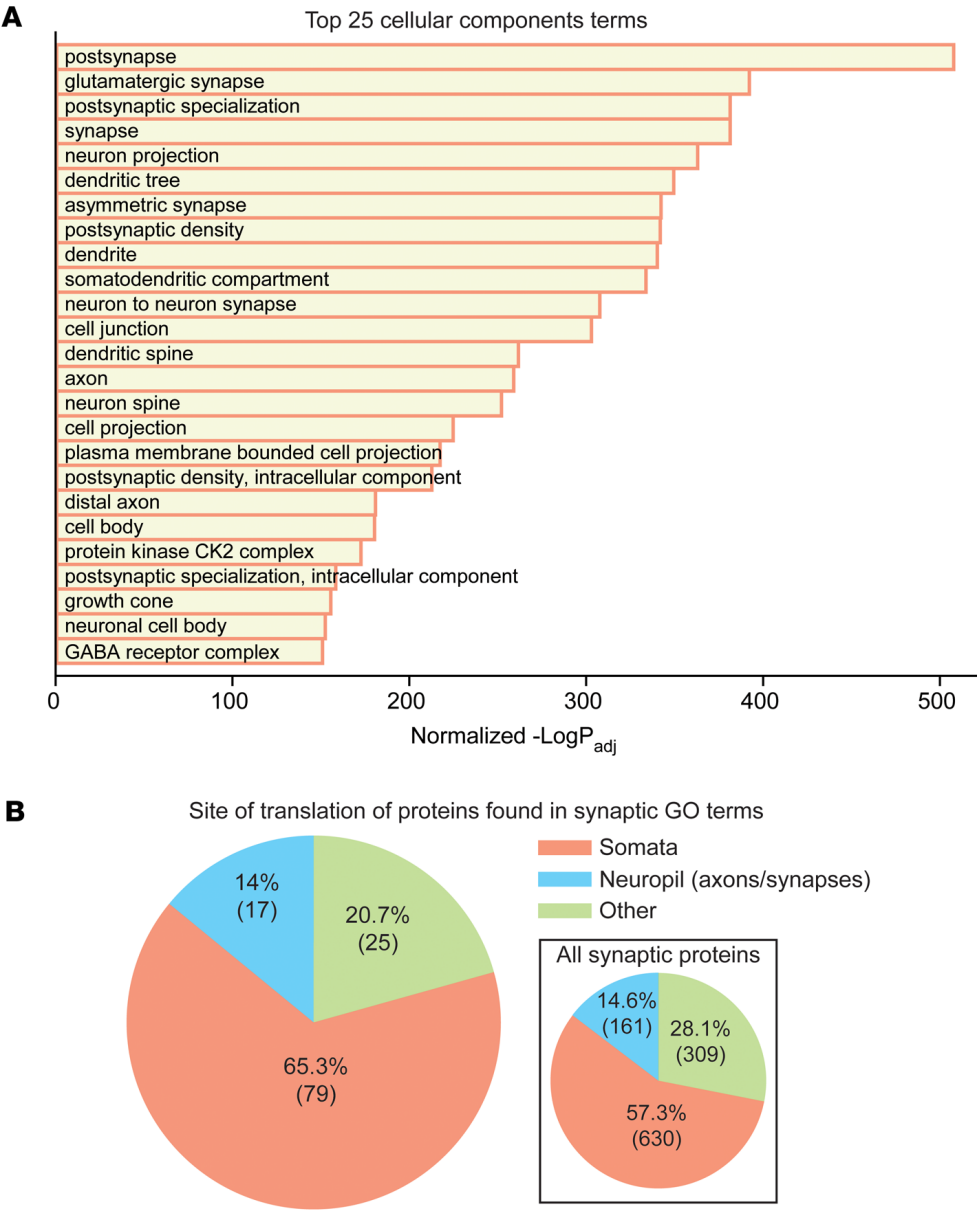
FGF13S is near soma-translated synaptic proteins. (**A**) Top 25 cellular component terms from gene set enrichment analysis showing a high representation of synaptic and distal axonal terms. (**B**) Pie chart showing the majority of the proteins found in the cellular component terms shown in **A** are translated in the soma and not locally at the synapses. Sites of translation were obtained from Glock et al. (51). Thirty-two proteins were not present in the translation database; therefore, 131 proteins were annotated. Inset: site of translation of all synaptic proteins, defined as the 2,025 proteins comprising the *Mus musculus* GO:0045202 term synapse. A total of 925 proteins are not present in the translation database; therefore, 1,100 proteins were annotated.

We first validated the TurboID-FGF13S expression in isolated hippocampal neurons by staining for HA (Supplemental Figure 3). The transgene expression pattern recapitulates the somatic and AIS enrichment of endogenous FGF13S (23), albeit with an overall stronger signal due to overexpression. After in vivo biotin injections (or vehicle control) to promote TurboID-FGF13S labeling of near neighbors, we captured biotinylated proteins with streptavidin and quantified them by mass spectrometry (MS). We detected robust protein labeling in brains from mice injected with biotin compared with DMSO-injected mice (Supplemental Figure 4A). Quantitative MS analysis detected 1,568 proteins (Supplemental Dataset 1 and Supplemental Figure 4B), 457 of which were significantly enriched in biotin-injected samples [*P* < 0.05 and log_2_(biotin/DMSO) > 1] (Figure 2A). Among the most enriched proteins was Na_V_1.6 (encoded by *Scn8a*), consistent with FGF13S’s known concentration in the AIS (10) and interaction with VGSC cytoplasmic C-termini (13, 14). Other AIS enriched proteins, such as ankyrin G (*Ank3*) and β4 spectrin (*Sptbn4*); the potassium channels KCNQ2 (*Kcnq2*), K_V_2.1 (*Kcnb1*), and K_V_2.2 (*Kcnb2*); MAP7D2 (*Map7d2*); and MICAL3 (*Mical3*) (39, 40), were prominently enriched in the dataset (Figure 3A). Other prominent AIS proteins such as gephyrin (*Gphn*), contactin (*Cntn1*), synaptopodin (*Synpo*), α2 spectrin (*Sptan1*), and PRICKLE2 were also identified, although at slightly above the *P* < 0.05 threshold. Sodium channel regulatory subunit β1 (*Scn1b*), a VGSC β subunit to which secreted FGF13S is proposed to cluster around via LRRC37B binding (27), was not detected.

With this validation of the dataset, we then asked whether any candidate FGF13S near-neighbors are secreted proteins, which could suggest that FGF13 is similarly processed for secretion, despite its lack of a signal peptide. We exploited a recent study by Uhlen et al. in which they identified the human secretome (41), compiling a list of all proteins that are secreted either extracellularly or intracellularly. The latter represents proteins going through the ER-Golgi canonical pathway but retained in intracellular organelles or vesicles. Of the 457 proteins significantly enriched in the FGF13S TurboID dataset, 14 (3%) are present in the secretome database, and only 2 (APOB and BTBD17) are designated as extracellularly secreted (we excluded proteins predicted to be secreted intracellularly or that have an unknown location) (Figure 2B). To assess the relevance of identifying 3% of proteins in the secretome database, we performed a similar analysis on a TurboID dataset for a known nonsecreted protein, the cardiac sodium channel Na_V_1.5, obtained in cardiomyocytes (42). Among the 1,015 proteins identified as Na_V_1.5 near neighbors, 50 (4.9%) were also present in the secretome database and 12 were annotated as secreted extracellularly. Thus, the 3% of proteins in the FGF13 TurboID dataset identified in the secretome appear to be within the range expected for nonspecific detection of secreted proteins. In contrast, the overlap between a different TurboID dataset designed to detect proteins secreted from various cell types (36) and the human secretome dataset was 48.7% (Figure 2B).

Additionally, we performed gene set enrichment analysis on the candidate FGF13 near neighbors and found that Gene Ontology (GO) terms with “secretion” were not among the top 100 biological processes identified (Supplemental Dataset 1). Furthermore, for those GO terms with the word secretion in their identifiers, there were 52 members (proteins) detected by TurboID. Analysis of these 52 proteins showed all were annotated as cytoplasmic or nuclear and none as secreted proteins (Supplemental Figure 5). We also noted that 2 key proteins necessary for the unconventional secretion of FGF2, TEC kinase and ATP1A1, are not among the significantly enriched FGF13S neighbors. Because FGF13S (like all FHFs) lacks a signal sequence, and secretion of the homologous FGF12 was proposed to use this unconventional pathway, the absence of TEC kinase and ATP1A1 among the candidate FGF13S near neighbors suggests FGF13S is unlikely to be secreted in a FGF2-like fashion. Rather, the 52 candidate FGF13S near neighbors found in GO terms with “secretion” in their identifiers are most likely cytoplasmic proteins that participate in secretory or other types of vesicle formation. Indeed, analysis of GO terms for biological processes or cellular components identified multiple terms containing “vesicle” among the top hits (Supplemental Figure 6). In a recent study of the related FGF13-VY in cardiomyocytes, we found that FGF13VY regulated trafficking of Cx43 (*Gja1*) containing vesicles via affecting microtubule stability (42). Thus, FGF13S may perform similar vesicle-trafficking roles in neurons, and the identification of FGF13 in LEV and SEV fractionation experiments (Figure 1) is consistent with a role of FGF13 in regulating vesicle formation and/or vesicle trafficking.

## Discussion

Although FHFs are members of the FGF superfamily, of which almost all members are proteins secreted through the conventional pathway and activate growth factor receptors through interaction with the receptors’ extracellular ligand binding domains (43), the initial report identifying FHFs noted the absence of signal sequences and explicitly reported that FGF12 (FHF1) was not secreted into the extracellular space (38). Subsequent studies identified various cytoplasmic FHF binding partners, including the cytoplasmic C-terminal domain of various VGSCs (2, 3, 12), the MAP kinase scaffold IB2 (44), microtubules (16), junctophilin 2 (45), and cavins (46). Lately, several studies have challenged the hypothesized restriction of FHFs to the cytoplasm, suggesting FHFs (specifically, FGF12A and FGF13S) can be secreted extracellularly, where they locally modulate biological processes by binding to the extracellular domains of integral membrane proteins (24, 25, 27). Although FGF13S has been extensively characterized as a regulator of VGSCs via binding to the channels’ intracellular C-terminal domain (47) and, more recently, by affecting local membrane cholesterol content (48), Libe-Philippot et al. reported FGF13S was secreted from neurons and bound to the extracellular domain of the human-specific protein LRR37B, resulting in modulation of Na_V_1.6 sodium channels at the AIS (27). In that study, however, results supporting FGF13 as an extracellular protein relied solely on the detection of FGF13S in the extracellular medium after expression of FGF13S in HEK293 cells, albeit without using specific steps to eliminate FGF13S released from dead or dying cells or FGF13S within extracellular vesicles. Furthermore, in the absence of comparison to a validated secreted protein, the relative efficiency of FGF13S secretion could not be assessed. Whether FGF13S is secreted from neurons was not tested.

Here, we tested if FGF13S can be secreted from HEK293 cells using an analogous experimental setup with validated positive and negative controls and adding differential centrifugation steps to reduce contamination from apoptotic cells and separate EVs. We were unable to detect secretion despite finding abundantly expressed adipsin, a conventionally secreted protein. Rather, differential centrifugation showed some FGF13S resides within LEVs and SEVs. We also tested if FGF13S is secreted from cultured hippocampal neurons. Although we detected robust secretion of clusterin, a positive control for an endogenously secreted protein, we observed no endogenous FGF13 isoform secretion, including FGF13S. Furthermore, by analyzing a database of proteins detected as FGF13S near neighbors via TurboID, we obtained no evidence of FGF13S secretion. Thus, with positive and negative controls, our results suggest FGF13S is not available to bind LRRC37B and thereby regulate Na_V_1.6 from the extracellular side of the membrane, as proposed (27). We conclude that FGF13S remains in the cytoplasm.

Although the Libe-Philippot et al. study showed regulation of neuronal excitability after application of purified FGF13S (or the N-terminal peptide encoded by the S exon), the biological relevance of those results is unclear if FGF13S is not secreted. Additionally, those experiments did not use negative controls (e.g., a scrambled peptide). Whereas here we specifically focused on the FGF13S isoform, our use of a pan-FGF13 antibody in the cultured hippocampal neuron experiments suggests that none of the FGF13 splice variants in the mouse brain are secreted.

Beyond its well-characterized regulatory effects on VGSCs, recent work we conducted, as well as from others, showed that FGF13 affects Κ^+^ channels (23, 49), controls local membrane cholesterol (42), stabilizes microtubules (16, 42, 50), and traffics ion channels and gap junctions to the plasma membrane (42, 48). Our results add to previous reports showing that FGF13 controls ion channel trafficking and suggest a potentially broader role in trafficking. Specifically, gene set enrichment analysis revealed that FGF13S appears to be a near neighbor of an unexpectedly large quantity of synaptic proteins. Synapse is the top among the GO cellular components terms and comprises 153 of all the proteins identified (33.5%). Several synapse-related terms, both pre- and postsynaptic, are among the top 25 terms (Figure 3A). Yet previous studies and data we report here, including immunofluorescence staining of both endogenous and TurboID-tagged FGF13S (Supplemental Figure 3), have not shown FGF13 to reside at either pre- or postsynaptic membranes. We hypothesize the synapse-related terms likely result from a role for FGF13 in trafficking of synaptic proteins to the synapse. Consistent with our hypothesis, we annotated synaptic proteins identified as FGFG13 near neighbors as predominantly translated either in the somatic compartment, in neurites, or “other,” based on a previous dataset (51), and we found that 65.3% were translated in the soma compared with 14.0% that were labeled as translated in neurites (20.7% were “other”). Thirty-two synaptic proteins among the FGF13 near neighbors were not present in that dataset, so 131 of 153 proteins were analyzed. When compared with the sites of translation of all synaptic proteins, as defined by the GO term synapse (GO:0045202), the distributions in our dataset were similar (somata: 57.3%; neuropil:14.6%; other: 28.1%) (Figure 3B, inset). If FGF13S localized to the synapse, the fraction of neuropil-translated proteins should be higher. Thus, most synaptic proteins identified as FGF13S near neighbors appear to be predominantly translated in the soma before being translocated to the synapse (Figure 3B). This conclusion is consistent with an expanding list of roles for cytoplasmic roles for FGF13. We note that, similar to the FGF13S dataset, 925 of 2,025 synaptic proteins are not present in the Glock et al. dataset (51). This is likely due to the different organisms (i.e., mouse in the present study; rat in the Glock et al. study, ref. 51) and different brain regions (whole brain with expression driven by *Nefh* promoter in the present study; the CA1 region of the rat hippocampus in the Glock et al. study, ref. 51).

Unexpectedly, we detected FGF13S within LEVs and SEVs released from HEK293 cells. This was not dependent on stress, because EVs isolated from both normal and serum-deprived medium showed similar results (Figure 1A). Although we attempted to remove cell debris by centrifugation to reduce contamination from FGF13S released by dead cells, we cannot rule out that some of the detected FGF13S in the LEV pool was due to its presence in apoptotic vesicles, because the LEV pool also contains other types of large vesicles, such as large ectosomes and oncosomes. Although we did not detect any FGF13 isoform in EVs from cultured mouse hippocampal neurons (Figure 1B), its presence in EVs might depend on several factors, including cell type, tissue, stimuli, and/or pathophysiological changes. Further studies are indicated to better understand the possible role of FGF13 and potentially other FHFs in LEVs. The SEV pool (which includes exosomes, approximately 30–150 nm vesicles that play crucial roles in cellular communications) has been the subject of substantial focus due to exosomes’ tumorigenic roles (52). Further studies may clarify the presence and role of FGF13 in exosomes, but this finding is intriguing because FHFs, particularly FGF13, have been implicated in different types of cancer (53–56).

A separate study suggested FGF12 can be secreted from cells using an FGF2-like unconventional secretion pathway in which FGF2 is first recruited to the plasma membrane via interaction with sodium-potassium ATPase (specifically subunit ATP1A1) and is then phosphorylated by TEC kinase and subsequently oligomerizes with other FGF2 molecules and undergoes self-secretion (24). Our assays would detect FGF13S secretion from such an unconventional pathway. Furthermore, because we detected neither sodium-potassium ATPase nor TEC kinase in the FGF13S TurboID dataset, we conclude that FGF13-S is not secreted via the FGF2-like mechanism.

In conclusion, this study shows that FGF13 is not secreted from HEK293 cells or mouse hippocampal neurons. Although we detected some FGF13 in extracellular vesicles from HEK293 cells by using differential centrifugation—and FGF13 in EVs could explain the reported FGF13 detected in the extracellular medium of HEK293 cells in a previous study (27)—we note that FGF13 within EVs would be incapable of binding LRR37B extracellularly. Thus, our results suggest modulation of VGSCs and neuronal excitability depends only upon the cytoplasmic functions of intracellular FGF13S.

## Methods

### Sex as a biological variable.

Male and female mice were used in this study. Sex was not considered as a biological variable. The only exception applies to the generation of *Fgf13* neuronal-specific knockout mice for the whole-brain lysates shown in Figure 1B. *Fgf13* is X-linked, and we were limited to studying hemizygous males (*Nes-cre;Fgf13^fl/Y^*) because whole-body or neuronal knockout (with Nestin-Cre) is lethal before weaning (23, 57, 58), thus precluding the viability of mature hemizygous male knockouts necessary for generating female homozygous knockouts.

### HEK293 cell cultures.

HEK293 human embryonic kidney cells (ATCC, CRL-1573) were grown and maintained in a 5% CO_2_ incubator at 37°C in DMEM (Gibco, 11995073) supplemented with 10% FBS (VWR, 97068-085) and 1% penicillin-streptomycin (Gibco, 15140122).

### Plasmids and HEK293 cell transfection.

*hFGF13S* cDNA was cloned in a pcDNA3.1 background (7). The murine adipsin-HA expression plasmid (59) was provided by James Lo (Weill Cornell Medicine, New York, NY, USA). HEK293 cells were seeded in 6-well plates coated with poly-d-lysine (0.1 mg/mL; Gibco, A3890401) and transfected with Lipofectamine 2000 (Invitrogen, 11668019) according to the manufacturer’s instructions.

### Isolated mouse hippocampal neuron cultures.

Hippocampi were dissected from P0 newborn pups and dissociated through enzymatic treatment with 0.25% trypsin (Gibco, 15090046) and subsequent trituration. The cells were cultured in 6-well plates for secretion experiments or plated on glass coverslips previously coated with poly-d-lysine (0.1 mg/mL; Gibco, 3890401) and laminin (20 μg/mL; Gibco, 23017015) in 24-well cell culture plates for immunofluorescence experiments. The hippocampal cells were grown in Neurobasal A medium (Gibco, 10888022) supplemented with 2% B-27 (Gibco, 17504044), 2 mM l-glutamine (Gibco, 25030081), 10% FBS (VWR, 97068-085), and 1% penicillin-streptomycin (Gibco, 15140122) in a 5% CO_2_ incubator at 37°C overnight. After 24 hours, the medium was replaced by culture medium containing 2% B-27, 0.5 mM glutamine, 1% FBS, 70 μm of uridine (Millipore Sigma, U3003), and 25 μm of 5-fluoro-2′-deoxyuridine (Millipore Sigma, F0503) and cultured in a 5% CO_2_ incubator at 37°C.

### Medium clearing and extracellular vesicle isolation.

Twenty-four hours after transfection, HEK293 cell medium was replaced with serum-free medium (DMEM supplemented with 1% penicillin-streptomycin) or normal medium (10% serum) and cells were incubated for 24 hours in a 5% CO_2_ incubator at 37°C. The medium was then collected and subjected to sequential centrifugation steps to clear it and isolate extracellular vesicles. First, it was centrifuged for 5 minutes at 4,000*g* on a tabletop centrifuge to remove cell debris. The supernatant was collected and centrifuged in an Optima XE-100 ultracentrifuge in a Ti rotor (Type 50.4; Beckman Coulter) for 20 minutes at 12,000*g* at 10°C to pellet LEVs. The supernatant was collected and then centrifuged for 70 minutes at 100,000*g* to pellet SEVs or exosomes. Cleared medium was collected and, for serum-deprived medium only, concentrated 30× in an Amicon ultracentrifugal filter (10 kDa MWCO; Millipore Sigma, UFC801024). Pellets were resuspended in 100 μL of RIPA buffer (150 mM NaCl; 50 mM Tris-HCl pH 7.4; 1% Triton X-100, v/v; 0.1% SDS, w/v; 0.5% sodium deoxycholate, w/v, 1 mM EDTA) supplemented with complete protease inhibitor cocktail (Roche, 11836170001) and 1 mM PMSF (ThermoFisher Scientific, 36978). Pellets and cleared medium were run on a gel at the percentages of total volume indicated in Figure 1A. Separately, cells were washed in PBS and lysed in RIPA buffer. Lysates were centrifuged at 17,000 *g* at 4°C for 15 minutes, supernatant was collected, and protein concentration was measured by Bradford assay (Pierce, 1863028).

Isolated mouse hippocampal neurons were cultured for 12 days in 6-well plates. The medium was then collected and centrifuged at 4,000*g* for 5 minutes on a tabletop centrifuge to remove cell debris. Separately, cells were washed in PBS and lysed in cold RIPA buffer. Lysates were centrifuged at 17,000*g* at 4°C for 15 minutes, supernatant was collected, and protein concentration was measured by Bradford assay (Pierce, 1863028). Medium and lysates were run on a gel at the percentages of total volume indicated in Figure 1B. For clusterin time-dependent secretion experiments, isolated mouse hippocampal neurons were cultured for 11 days in 6-well plates, after which the culture medium was replaced with fresh medium in half the wells and incubated for 24 hours, and the other wells remained untouched. The following day, medium was collected and cleared as described.

### Western blotting.

Samples were separated on Novex Tris-Glycine, 8%–16% polyacrylamide gels (Invitrogen, XP08160BOX) and transferred to PVDF membranes using the iBlot 3 Western Blot Transfer System (ThermoFisher Scientific). Membranes were blocked in blocking buffer (5% BSA, w/v; 0.1% Tween 20, v/v) for 1 hour at room temperature and incubated overnight with the following primary antibodies diluted in blocking buffer at 4°C: mouse anti–FGF13-S 1:350 (Anti-Pan-FHF-A [N235/22R]; Addgene, 190888); rabbit anti-HA 1:1,000 (anti-HA tag; Cell Signaling, 3724S); mouse anti-GAPDH 1:1,000 (ThermoFisher Scientific, MA5-15738); mouse anti-Alix 1:1,000 (Cell Signaling, 2171); rabbit anti-CD9 1:1,000 (Cell Signaling, 12174); rabbit anti–syntenin 1:1,000 (Abcam, ab315342); rabbit anti-calnexin 1:1,000 (Cell Signaling, 2679); rabbit anti-FGF13 1:1,000 (custom designed, YenZym); and mouse anti-clusterin 1:500 (Santa Cruz, sc-5289). The previously described custom anti-FGF13 antibody (7) has been validated against *Fgf13* knockout models (23, 46, 48).

Membranes were washed 5 times in TBST (150 mM NaCl; 20 mM Tris pH 7.6; 0.1% Tween 20, v/v) and incubated with the following secondary antibodies diluted in blocking buffer for 1 hour at room temperature: anti-mouse HRP 1:5,000 (Santa Cruz, sc-516102) and goat anti-rabbit HRP (Cell Signaling, 7074). Membranes were washed 5 times in TBST and imaged using a ChemiDoc Touch Imaging System (Bio-Rad).

### Generation of brain-specific TurboID-FGF13S transgenic mice.

*hFGF13S* cDNA was fused at the 3′ end of *HA(x3) tag-TurboID-V5 tag* cDNA to generate the *TurboID-FGF13S* construct that was then cloned into the modified tetracycline-inducible promoter vector (60, 61) to generate tetracycline inducible TurboID-hFGF13S transgenic mice on a *C57BL/6* (The Jackson Laboratory, 000664) background. These mice were crossed with brain-specific B6;C3-Tg(Nefh-tTA)8Vle/J (NtTA) mice (The Jackson Laboratory, 025397) (62) with tTA expression for brain-specific expression of the TurboID-FGF13S construct. While breeding, mice were fed doxycycline (200 mg/kg; BioServ S3888) to suppress transgene expression. After weaning, mice were fed normal diet to allow for transgene expression.

### In vivo biotinylation and sample preparation for MS.

Male and female *NtTA/TurboID FGF13S* double-transgenic mice, 4–8 months old, were injected daily for 3 consecutive days with Biotin (10 μL/g, subcutaneously, from a 2.4 mg/mL stock in PBS/DMSO 9:1) or 10% DMSO (in PBS). The mice were sacrificed to collect the brains 24 hours after the third injection. Whole-brain tissues were lysed with a handheld tip homogenizer in lysis buffer (50 mM Tris-HCl pH 7.5; 150 mM NaCl; 10 mM EDTA; 0.5% sodium deoxycholate, m/v; 1% Triton X-100, v/v; 0.1% SDS, w/v) supplemented with complete protease inhibitor cocktail (Roche, 11836170001) and 1 mM PMSF (ThermoFisher Scientific, 36978). Lysates were centrifuged at 17,000*g* at 4°C for 15 minutes, the supernatant was collected, and protein concentration was measured by Bradford assay (Pierce, 1863028). Biotinylation efficacy was confirmed by Western blot analysis with streptavidin-HRP (10 μg/mL; ThermoFisher Scientific, S911).

Brain lysates were incubated overnight at 4°C with 50 μL of streptavidin magnetic beads (Pierce, 88817), prewashed in lysis buffer, to pull down biotinylated proteins. The next day, beads were pelleted using a magnetic rack, resuspended in lysis buffer, and transferred to new microcentrifuge tubes. Beads were washed 3 times with lysis buffer and 3 times with PBS. Before the final bead pull down, 10% of the resuspended beads were collected for Western blot analysis and the rest submitted for MS analysis.

### MS analysis.

MS analysis was performed at the Weill Cornell Medicine Proteomics and Metabolomics Core Facility. The protein on beads was reduced with DTT, alkylated with iodoacetamide, and digested overnight with trypsin at 37°C. The digests were desalted by C18 Stage-tip columns. The digests were analyzed using a ThermoFisher Scientific EASY-nLC 1200 coupled on-line to a Fusion Lumos mass spectrometer (ThermoFisher Scientific). Buffer A (0.1% formic acid in water) and buffer B (0.1% formic acid in 80% acetonitrile) were used as mobile phases for gradient separation. A 75 μm × 15 cm chromatography column (ReproSil-Pur C18-AQ, 3 μm; Dr. Maisch GmbH) was packed in-house for peptide separation. Peptides were separated with a gradient of 5%–40% buffer B over 30 minutes and 40%–100% buffer B over 10 minutes at a flow rate of 400 nL/min. The Fusion Lumos mass spectrometer was operated in a data-independent acquisition (DIA) mode. MS1 scans were collected in the Orbitrap mass analyzer from 350 to 14,00 m/z at 120,000 resolution. The instrument was set to select precursors in 45 × 14 m/z–wide windows with 1 m/z overlap from 350–975 m/z for HCD fragmentation. The MS/MS scans were collected in the orbitrap at 15,000 resolution. Data were searched against the mouse Uniprot database (downloaded on August 7, 2021) using DIA-NN, version 1.8, and filtered for a 1% false discovery rate for both protein and peptide identifications. Statistical significance was determined by multiple *t* tests adjusted for multiple comparisons.

### GO analysis.

GO analysis for proteins with a log_2_(biotinylated/control) > 2, adjusted *P* value (*P*_adj_) < 0.05, was performed using g:Profiler (https://biit.cs.ut.ee/gprofiler/gost) (32). For each GO term, we first computed the gene ratio by dividing the Intersection_Size value by the Query_Size value. We then divided the gene ratio by (Term_Size / Effective_domain_size) to derive the enrichment ratio. We sorted the GO terms by the product of –log_10_ × *P*_adj_ and the enrichment ratio.

### Immunofluorescence.

Neurons were fixed after 12 days in culture in 4% paraformaldehyde (Millipore Sigma, 1588127) for 15 minutes, washed 3 times with PBS, and blocked/permeabilized in 2.5% BSA (Millipore Sigma, A9418) with 0.2% Triton X-100 (Millipore Sigma, T8787) in PBS for 1 hour at room temperature. Coverslips were incubated overnight with the following primary antibodies diluted in 2.5% BSA at 4°C: mouse anti–FGF13-S 1:250 (Anti-Pan-FHFA [N235/22R]; Addgene, 190888) and rabbit anti-HA 1:1,000 (anti-HA tag; Cell Signaling, 3724S). Coverslips were washed 5 times in PBST (0.1% Tween-20 in PBS; ThermoFisher Scientific, J20605.AP) and incubated with the following secondary antibodies diluted 1:500 in 2.5% BSA for 1 hour at room temperature: goat anti-mouse Alexa Fluor-488 (Invitrogen, A11001) and donkey anti-rabbit Alexa Fluor-568 (Invitrogen, A10042). Coverslips were washed 5 times in PBST, incubated for 5 minutes with DAPI at room temperature, washed 3 times in PBS, and mounted on glass slides (Matsunami, SUMGP11) with mounting medium (Vectashield, H1000). Images were collected using a Leica DMi8 fluorescent microscope with Thunder imager processing.

### Statistics.

Statistical parameters are reported in the figures and figure legends. Unpaired 2-tailed Student’s *t* tests (assuming equal variance) were used as the statistical method. Data, graphs, and images were generated with Microsoft Excel, ImageJ Fiji (63), ImageLab (BioRad), SigmaPlot, and Adobe Illustrator. *P* values of less than 0.05 were considered significant.

### Study approval.

Mice were handled in accordance with the ethical guidelines of the NIH’s *Guide for the Care and Use of Laboratory Animals* (National Academies Press, 2011). This study was approved by the Weill Cornell Medical Center Institutional Animal Care and Use Committee (protocol 2016-0042).

### Data availability.

TurboID proteomics data and full GO data are available in the Supplemental Dataset 1.

## Author contributions

MM, HZ, PT, DL, SOM, and GSP designed the research studies; MM conducted the experiments and acquired data, MM and GSP analyzed the data and wrote the manuscript. All authors discussed the results and contributed to the final manuscript.

## Funding support

This work is the result of NIH funding, in whole or in part, and is subject to the NIH Public Access Policy. Through acceptance of this federal funding, the NIH has been given a right to make the work publicly available in PubMed Central.

- National Heart, Lung, and Blood Institute grants R01HL160089 and R01HL177538 to GSP and SOM.
- National Cancer Institute grant CA218513 to DL and HZ.

## Supplementary Material

Supplemental data

Supplemental data set 1

Unedited blot and gel images

Supporting data values

## Version 1 11/25/2025

In-Press Preview

## Version 2 01/09/2026

Electronic publication
