# Supplementary material for: FGF13 is not secreted from mouse neurons
Source: JCI Insight. 2025 Nov 25;11(1):e195998. doi: 10.1172/jci.insight.195998 (PMC12890474; doi:10.1172/jci.insight.195998)
Supplement: Supplemental data [file jciinsight-11-195998-s141.pdf]

SUPPLEMENTAL FIGURES FOR

FGF13 is not secreted from neurons

Mattia Malvezzi[1], Haiying Zhang[2], Patrick Towers[1], David C. Lyden[2], Steven O. Marx[3], and Geoffrey S. Pitt[1,†]

[1]Cardiovascular Research Institute, Weill Cornell Medicine, New York, New York, USA

[2]Children's Cancer and Blood Foundation Laboratories, Departments of Pediatrics, and Cell and Developmental Biology, Drukier Institute for Children's Health, Meyer Cancer Center, Weill Cornell Medicine, New York, NY, USA.

[3]Division of Cardiology, Department of Medicine, and Department of Molecular Pharmacology and Therapeutics, Vagelos College of Physicians and Surgeons, Columbia University, New York, New York, USA

†Correspondence to:
Geoffrey S. Pitt
Cardiovascular Research Institute
Weill Cornell Medicine
413 E. 69th St.
Belfer Research Bldg 502
New York, NY 10021
Email: geoffrey.pitt@med.cornell.edu
Phone: 646-962-7641

A

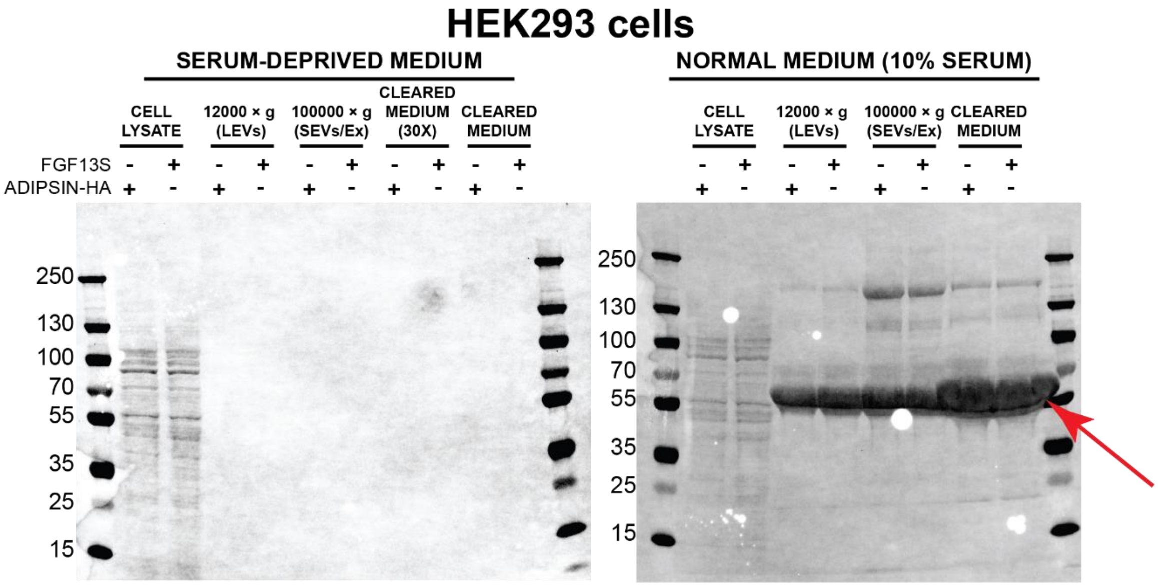

B

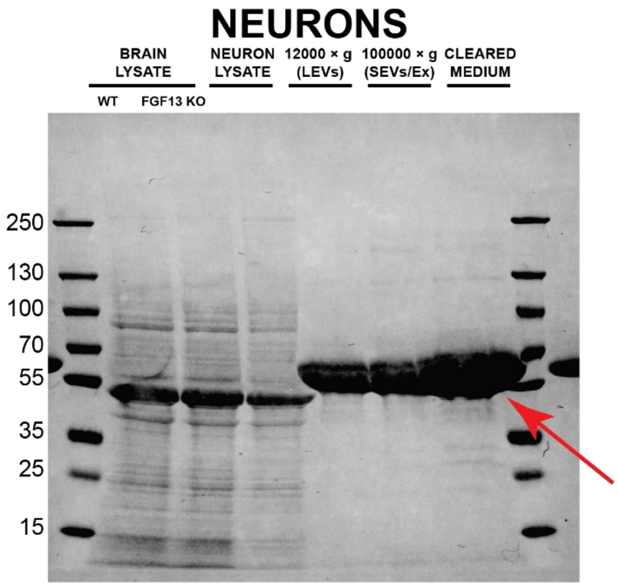

Supplementary Figure 1: Gel artifacts caused by serum and medium supplements. Ponceau-S staining of western blot membranes showing medium clearing experiments from HEK293 (**A**) and cultured mouse hippocampal neurons (**B**) of the blots shown in Fig. 1. In the presence of 10% serum (normal HEK293 culture conditions, **A**, right) and 1% serum and 2% B27 supplement (normal neuronal culture conditions, **B**) the high amounts of albumin and other supplements cause gel artifacts (red arrows) even without medium concentration. This precludes media concentration in comparison to serum-deprived conditions (A, left) where media is concentrated 30×.

A

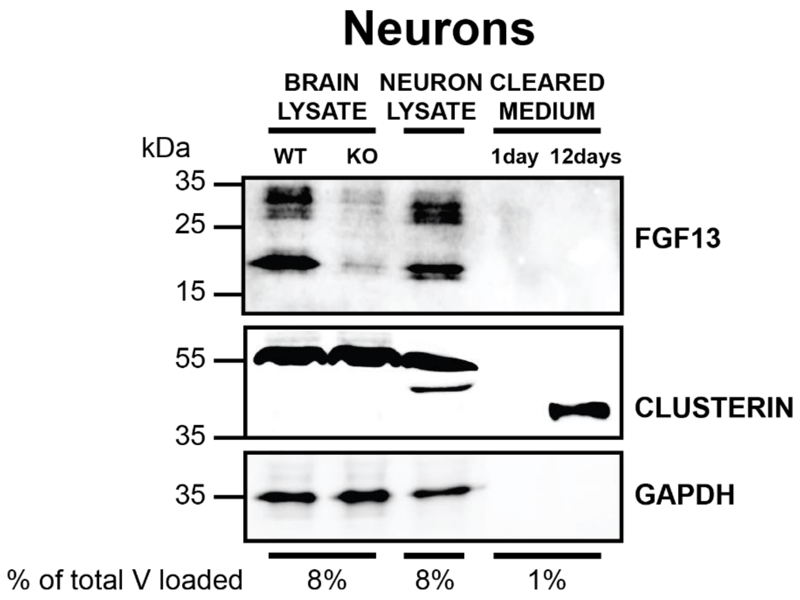

Supplementary Figure 2. Time-dependent secretion of clusterin in cultured neurons. Western blot of cell lysates and extracellular medium derived from cultured mouse hippocampal neurons. Medium was either left untouched for 12 days or changed the day before (11th day in culture). Whole brain lysates from wild-type (WT) and *Fgf13* brain knockout (KO) mice were used to validate the detection of FGF13 isoforms. GAPDH (36 kDa) is used as intracellular marker. Clusterin is used as a positive control for secretion in neurons, and it is only detected in cells cultured for 12 days but not 1 day, consistent with its time-dependent processing and secretion. 15 µg of brain or neuronal lysate were loaded. % of total V loaded indicates the fraction of the sample loaded on the gel relative to the total volume, allowing comparison among lanes.

A

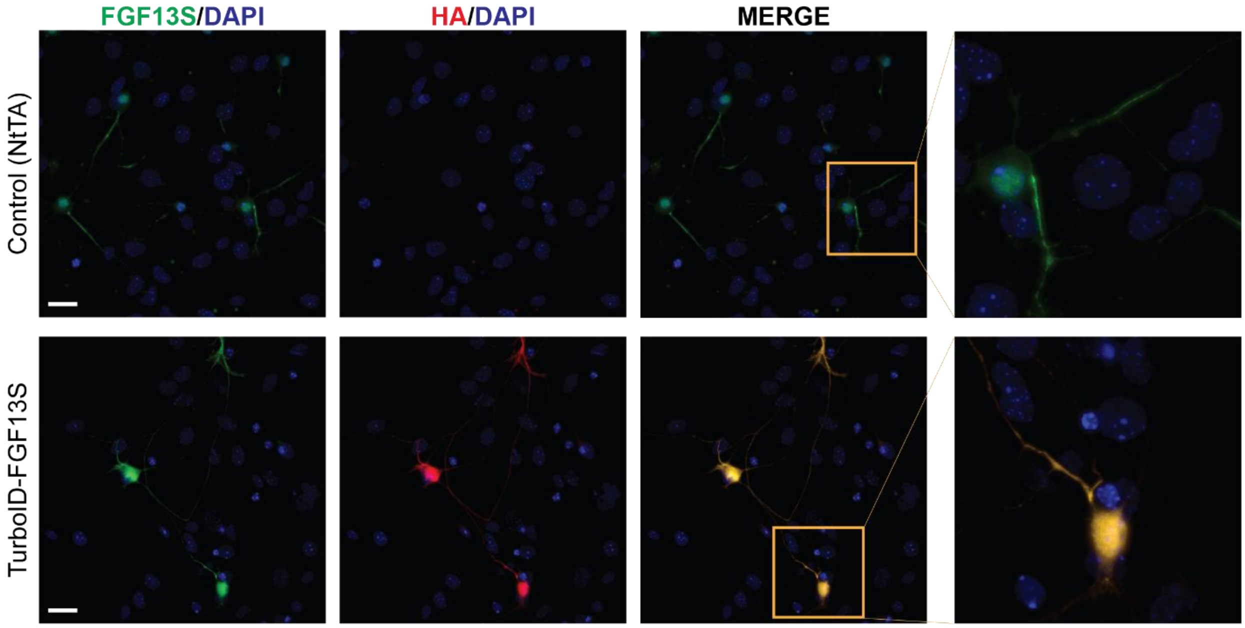

Supplementary Figure 3. Cellular localization of the TurboID-FGF13S transgene in mouse neurons.

A. Representative images from immunofluorescence staining of cultured hippocampal neurons isolated from control mice (NtTA background, top) and mice expressing the TurboID-FGF13S in the brain (bottom). Green: FGF13S; Red: Hemagglutinin (HA) tag. Blue: DAPI. Scale bars: 30 µm.

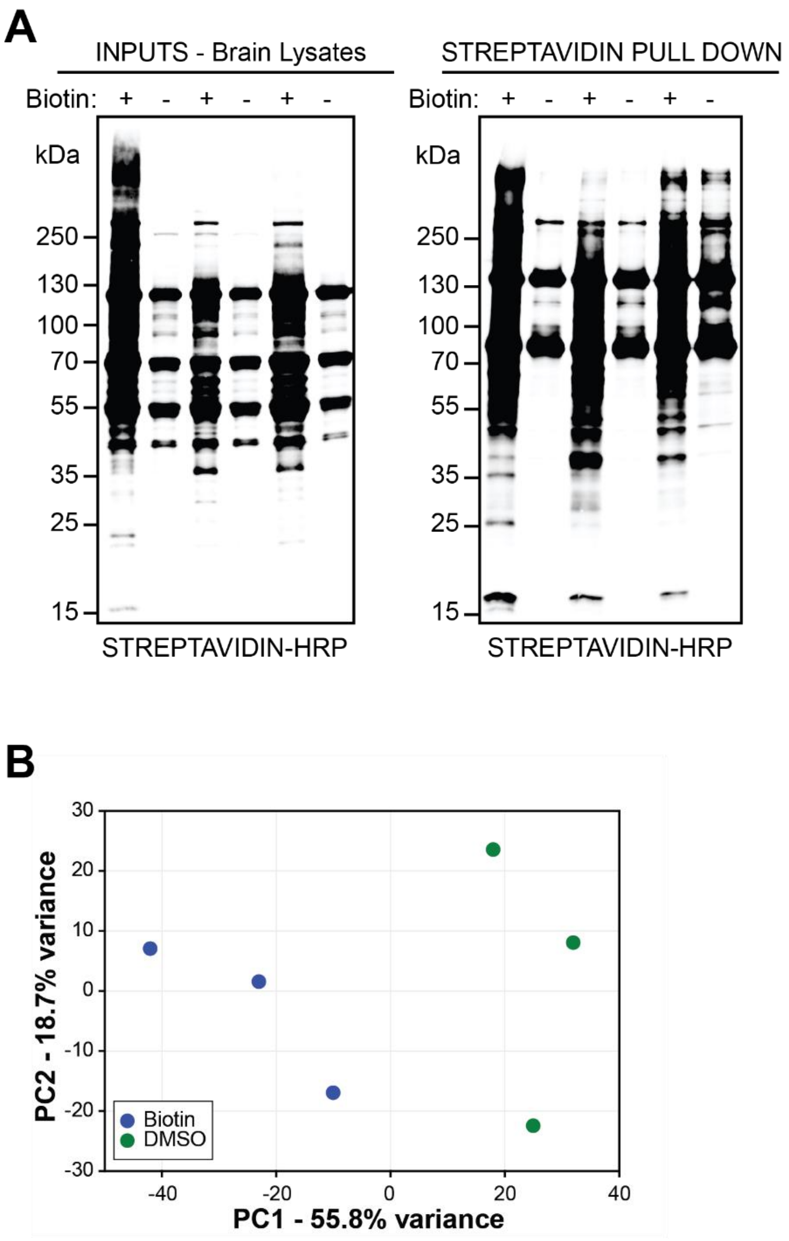

Supplementary Figure 4. *In vivo* labeling efficiency of TurboID-FGF13S in mouse brain.
A. Streptavidin-HRP blots of brain lysates and corresponding streptavidin pull-down samples from DMSO- or biotin-injected TurboID-FGF13S mice, showing robust biotin labeling. The same samples were then processed and analyzed by mass spectrometry.
B. Principal Component Analysis (PCA) plot from mass spectrometry analysis of samples shown in **A**.

A

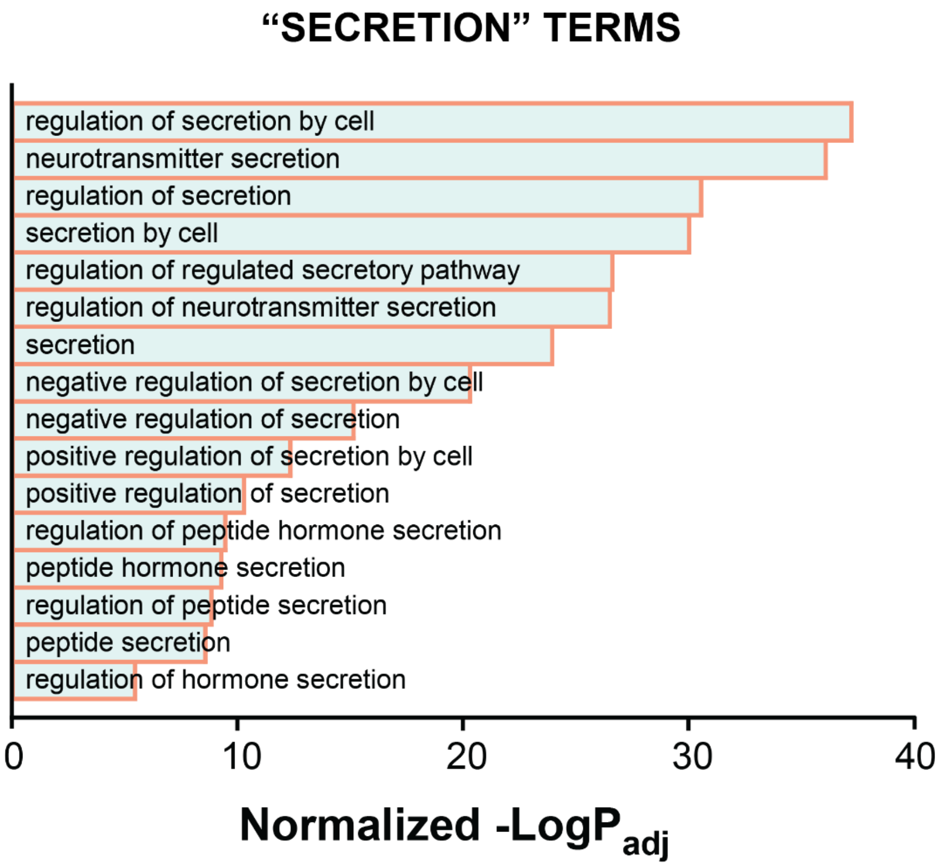

GENE	CYTOPLASMIC
Abr	YES
Akap5	YES
Arhgef7	YES
Atp9A	YES
Bcr	YES
Braf	YES
Brsk2	YES
C2Cd2L	YES
Cacna1A	YES
Cacnb4	NO (MEMBRANE)
Cadps	YES
Cbarp	YES
Cdk5	YES
Celsr2	YES
Coro1A	YES
Cplx2	YES
Dgki	YES
Dnm1L	YES
Dpysl2	YES
Dvl1	YES
Dynll1	YES
Exoc4	YES
Gabbr1	YES
Git1	YES
Gnaz	NO (MEMBRANE)
Grk2	YES
Hcfc1	YES
Hip1R	YES
Itsn1	YES
Kalrn	YES
Kcnb1	YES
Kcnc3	YES
Mical3	YES
Ncor2	NO (NUCLEAR)
Nf1	YES
Nos1	YES
Pdcd6lp	YES
Plcb1	YES
Ppp3Ca	YES
Prkaca	YES
Rab11Fip2	YES
Rab11Fip5	YES
Rab3Gap1	YES
Rtn4	YES
Septin5	YES
Snca	YES
Stim1	YES
Tardbp	YES
Trim9	YES
Vps18	YES
Vsnl1	YES
Ykt6	YES

Supplementary Figure 5. GO term analysis of "secretion" terms.
A. The full list of GO terms with "secretion" present in their identifiers. Right table shows cytoplasmic localization of all but one protein (NCOR2). *p*-value normalization details are provided in the *Methods* section.

A

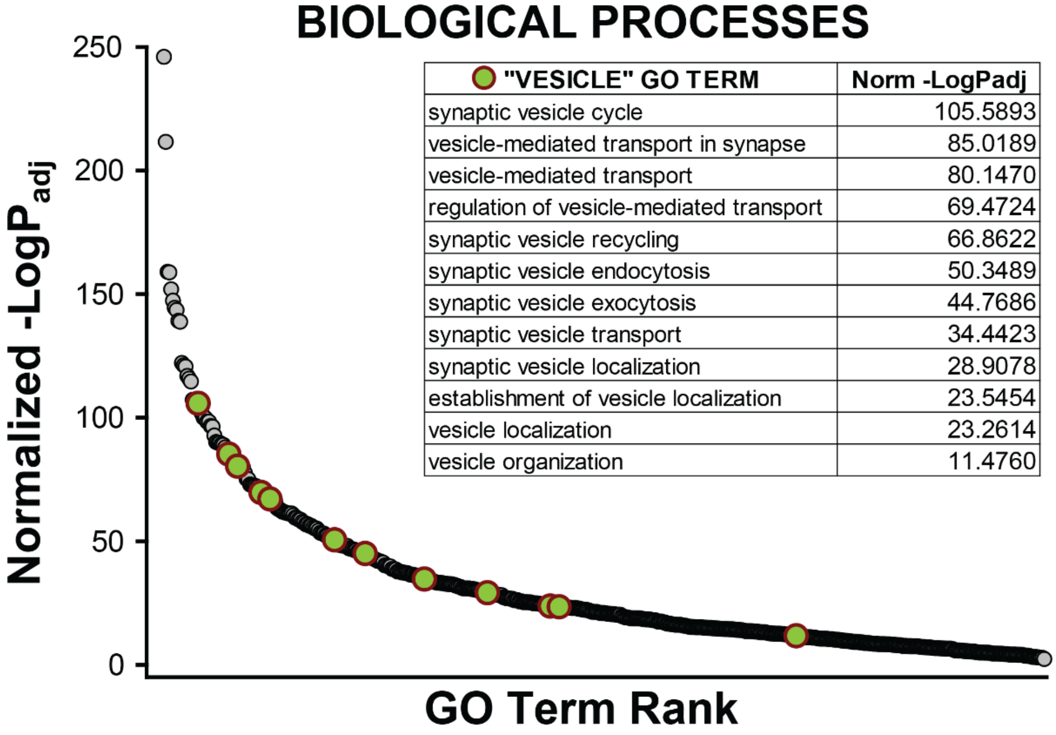

"VESICLE" GO TERM	Norm -LogPadj
synaptic vesicle cycle	105.5893
vesicle-mediated transport in synapse	85.0189
vesicle-mediated transport	80.1470
regulation of vesicle-mediated transport	69.4724
synaptic vesicle recycling	66.8622
synaptic vesicle endocytosis	50.3489
synaptic vesicle exocytosis	44.7686
synaptic vesicle transport	34.4423
synaptic vesicle localization	28.9078
establishment of vesicle localization	23.5454
vesicle localization	23.2614
vesicle organization	11.4760

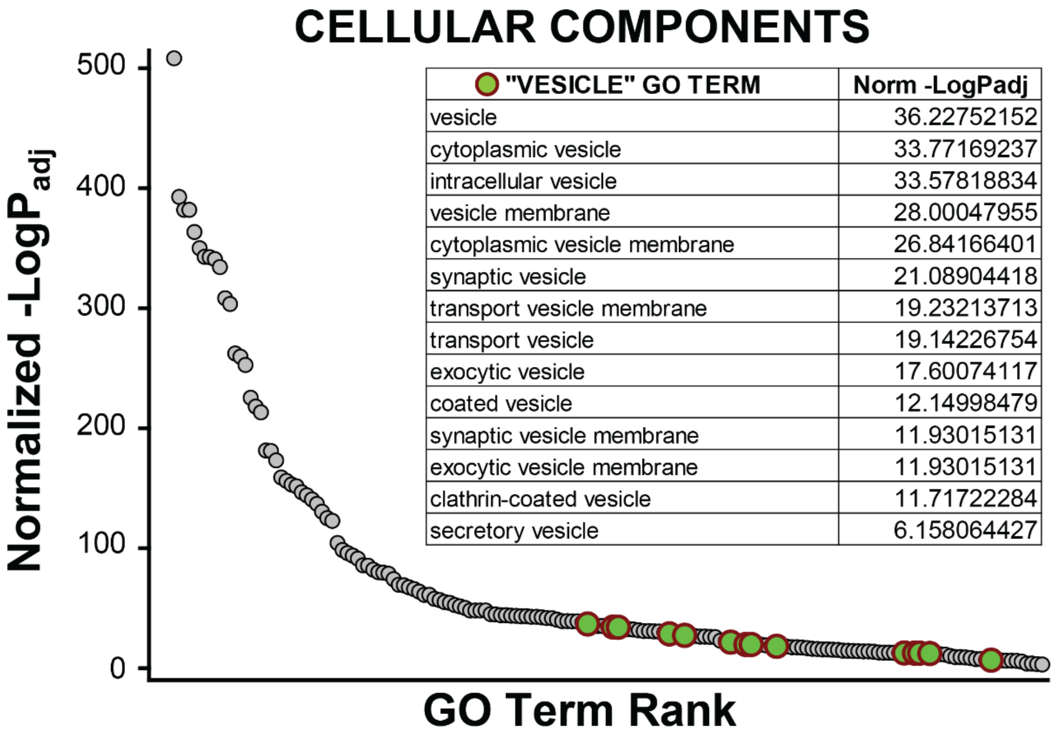

"VESICLE" GO TERM	Norm -LogPadj
vesicle	36.22752152
cytoplasmic vesicle	33.77169237
intracellular vesicle	33.57818834
vesicle membrane	28.00047955
cytoplasmic vesicle membrane	26.84166401
synaptic vesicle	21.08904418
transport vesicle membrane	19.23213713
transport vesicle	19.14226754
exocytic vesicle	17.60074117
coated vesicle	12.14998479
synaptic vesicle membrane	11.93015131
exocytic vesicle membrane	11.93015131
clathrin-coated vesicle	11.71722284
secretory vesicle	6.158064427

Supplementary Figure 6. GO term analysis of "vesicle" terms.
Biological Processes (**A**) and Cellular Components (**B**) GO terms (grey). Terms with "vesicles" in their identifiers are highlighted in green, and the full list is shown in the tables. The GO term rank is defined by the normalized -LogP_{adj} (descending). *p*-value normalization details are provided in the *Methods* section.
