# Supplementary material for: FGF13 is not secreted from mouse neurons
Source: JCI Insight. 2025 Nov 25;11(1):e195998. doi: 10.1172/jci.insight.195998 (PMC12890474; doi:10.1172/jci.insight.195998)
Supplement: Unedited blot and gel images [file jciinsight-11-195998-s143.pdf]

Uncropped blots – HEK293 cells serum-deprived (Fig.1A, left)

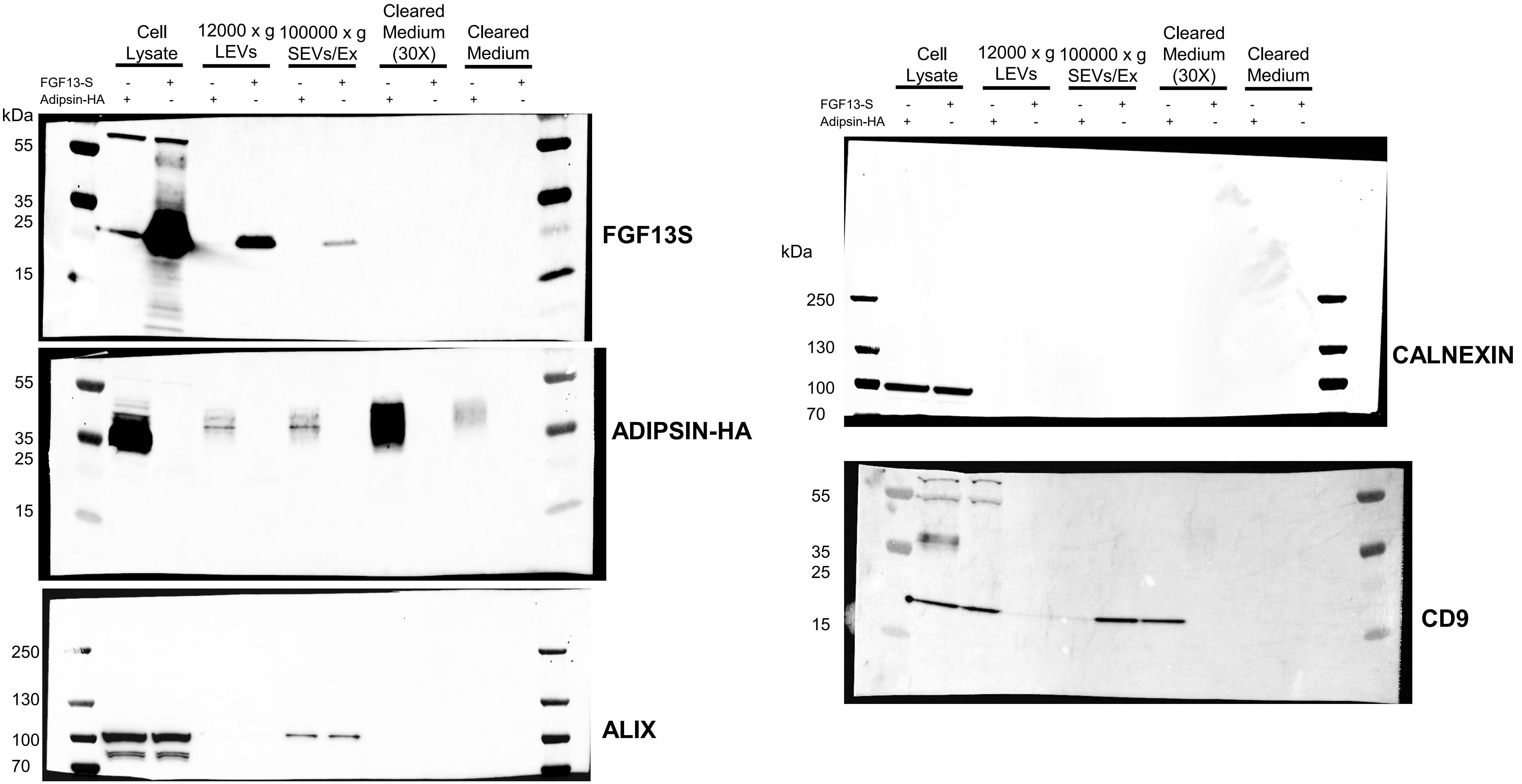

Uncropped blots – HEK293 cells 10% serum (Fig.1A, right)

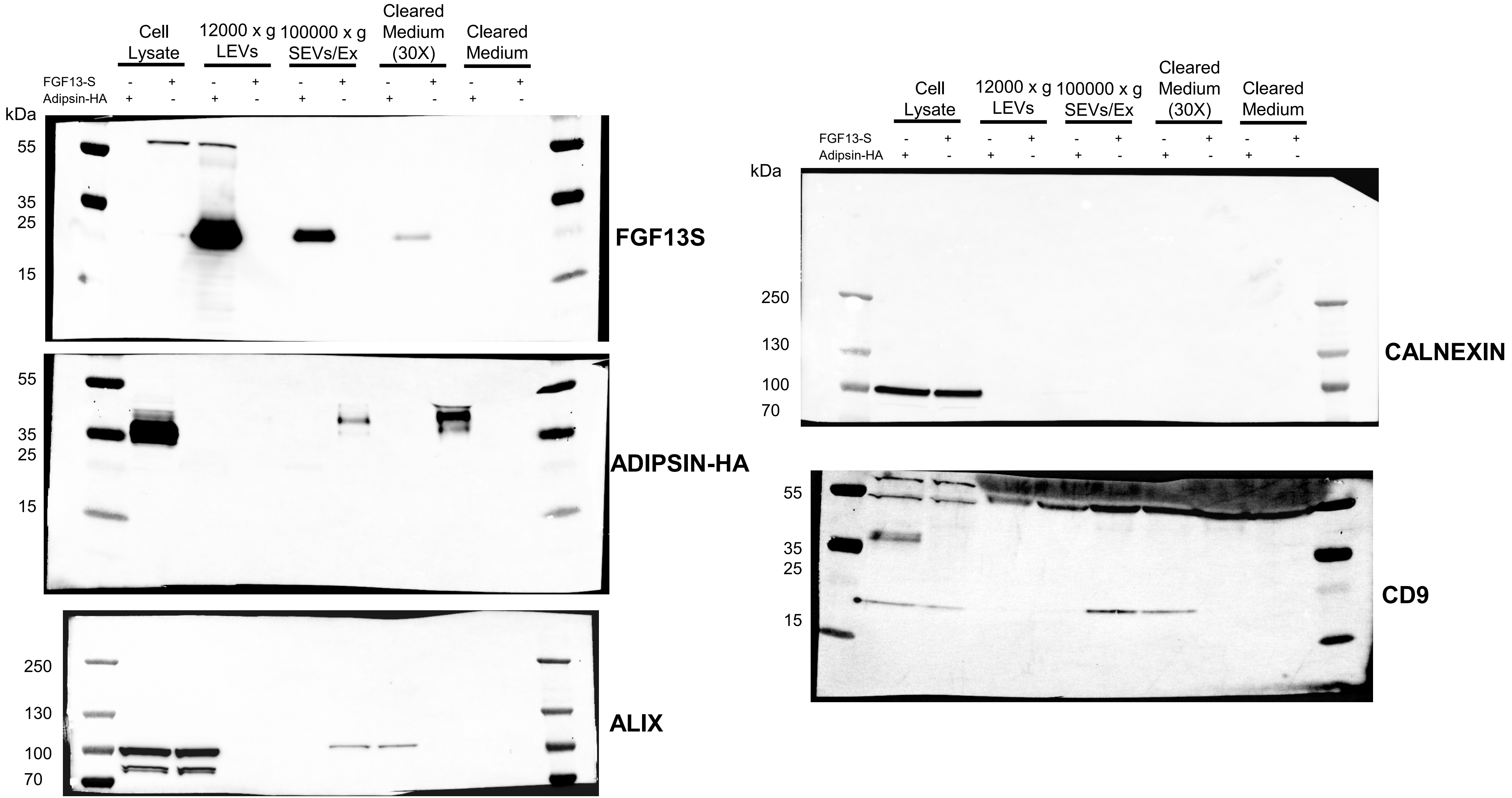

Uncropped blots – Neurons (Fig.1B)

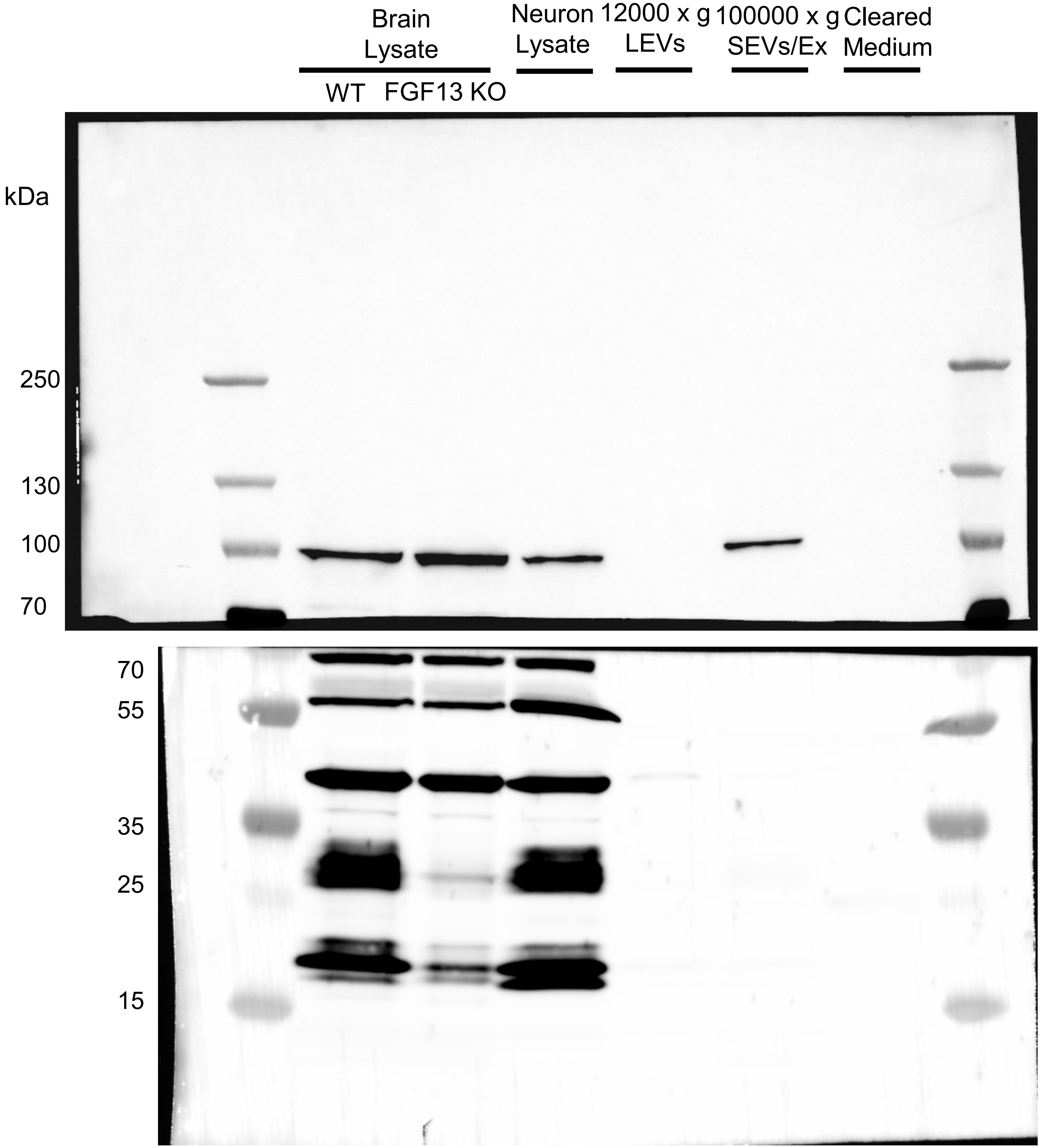

ALIX

FGF13 (pan)

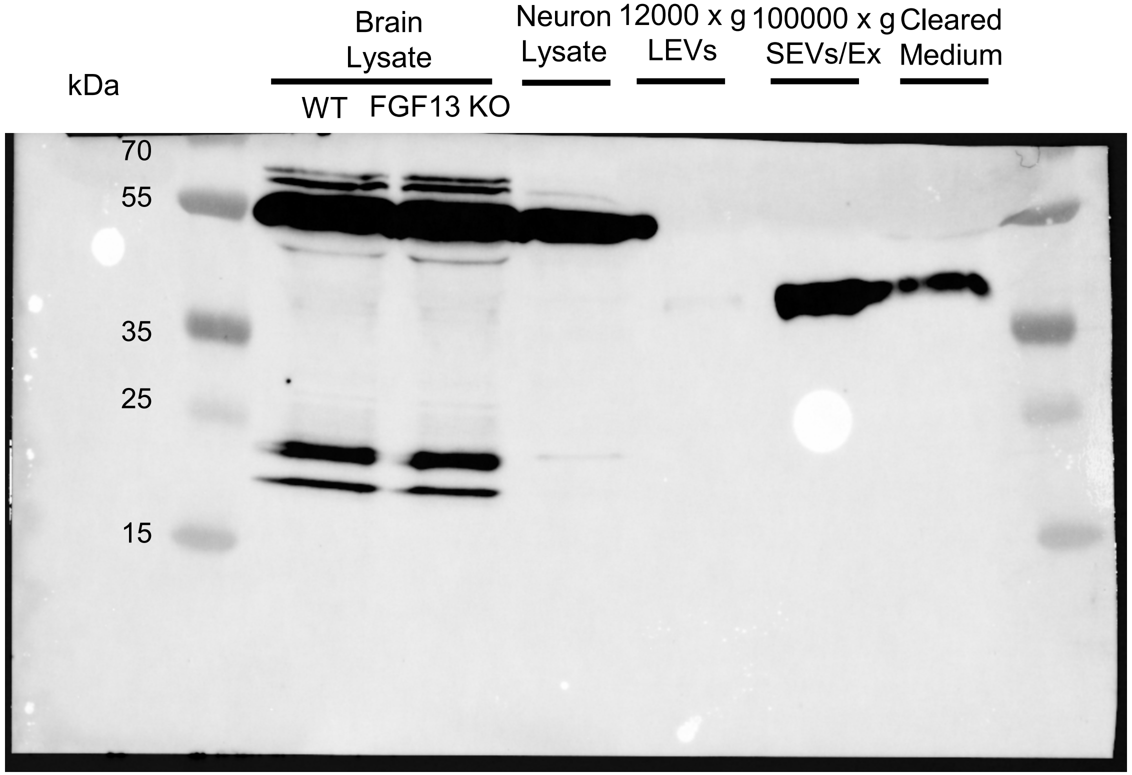

CLUSTERIN

Uncropped blots – Neurons (Fig.1B)

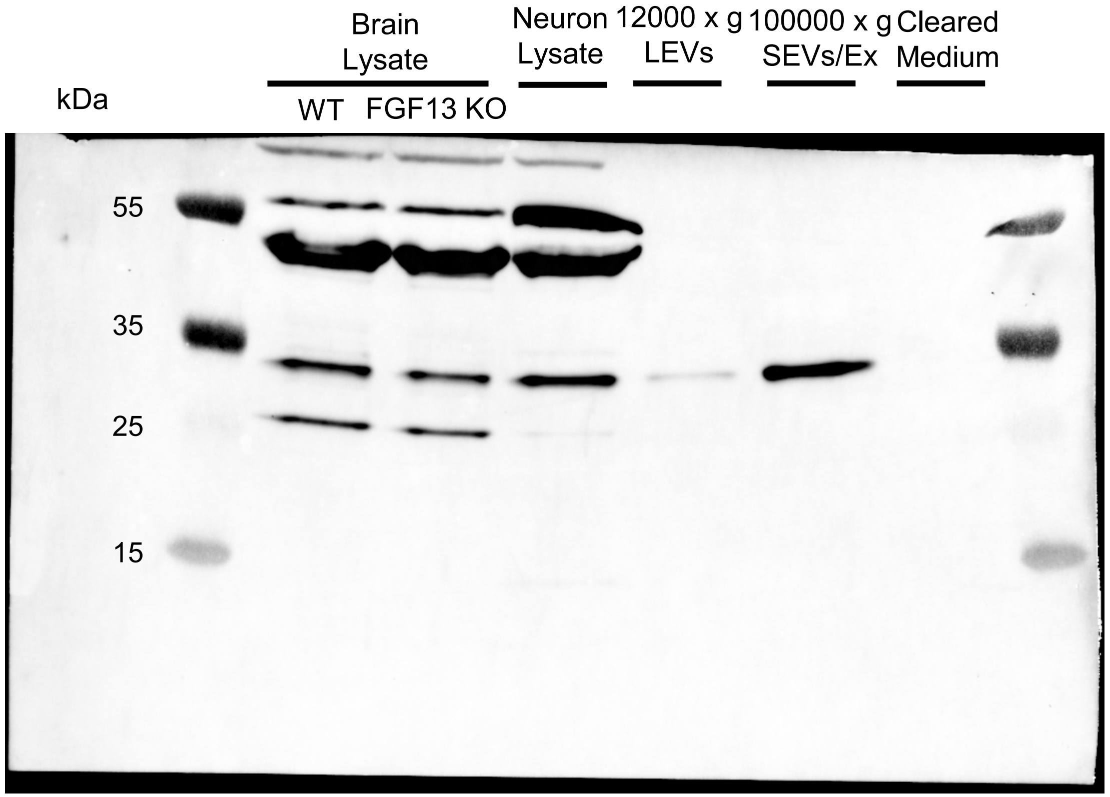

SYNTENIN

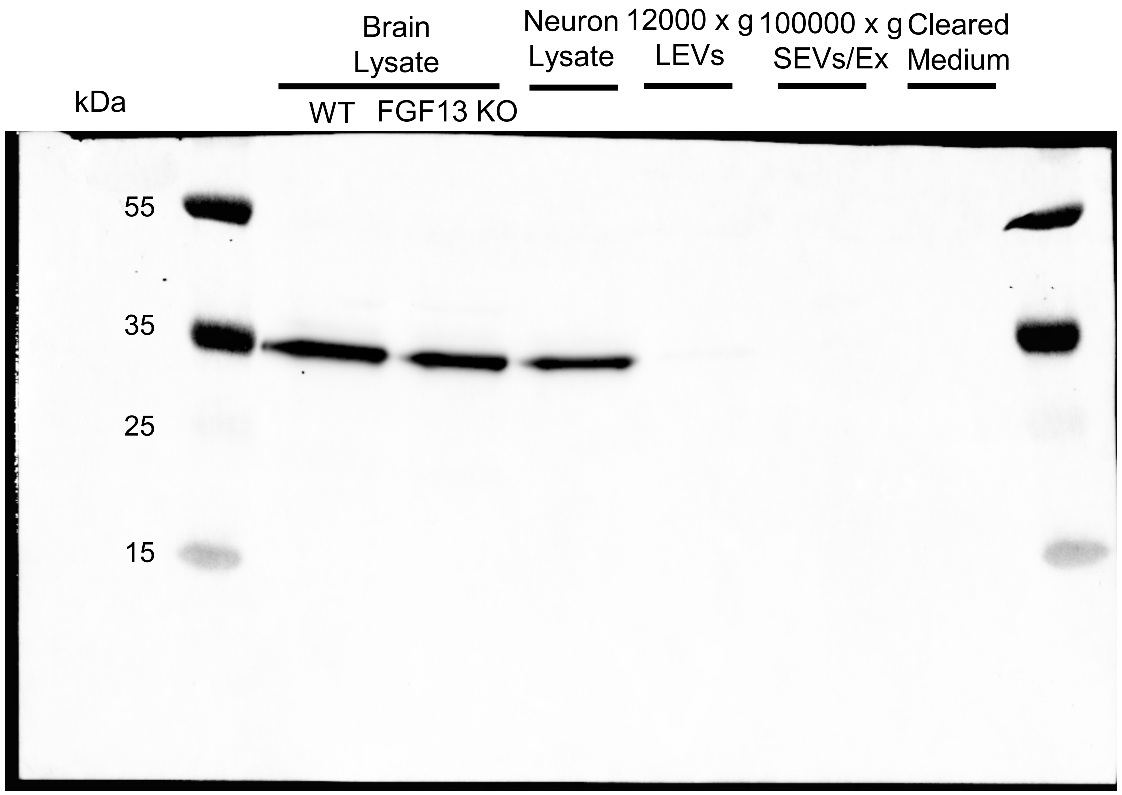

GAPDH

Uncropped blots – Neurons (Suppl. Fig. 2)

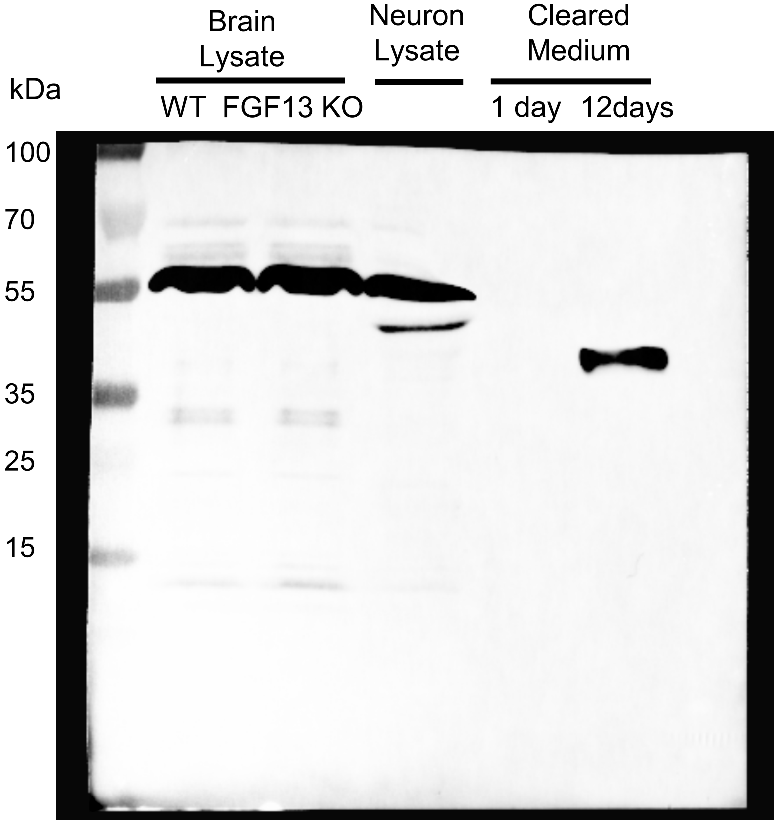

CLUSTERIN

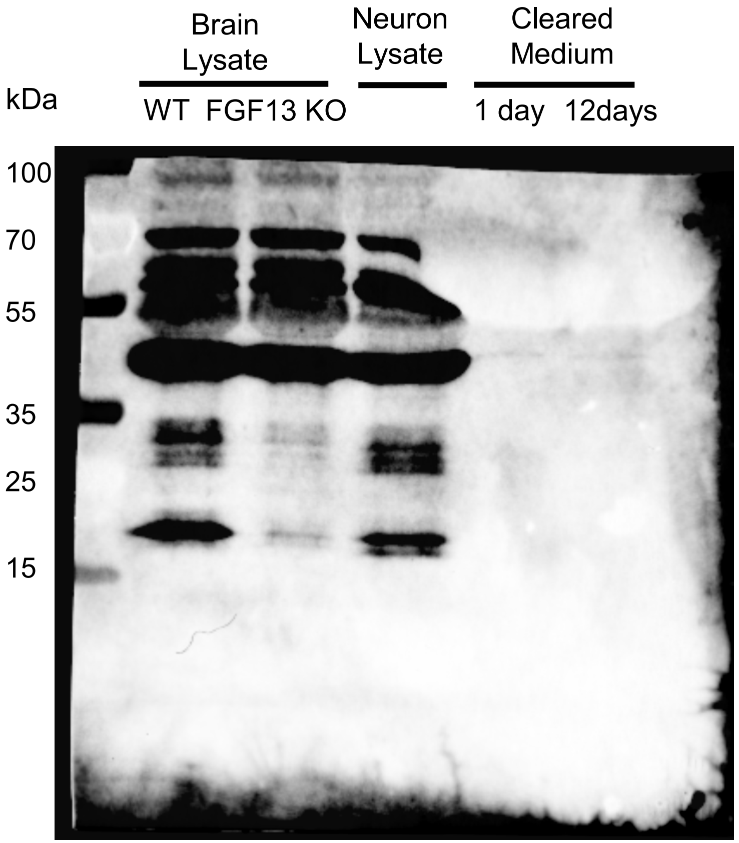

FGF13 (pan)

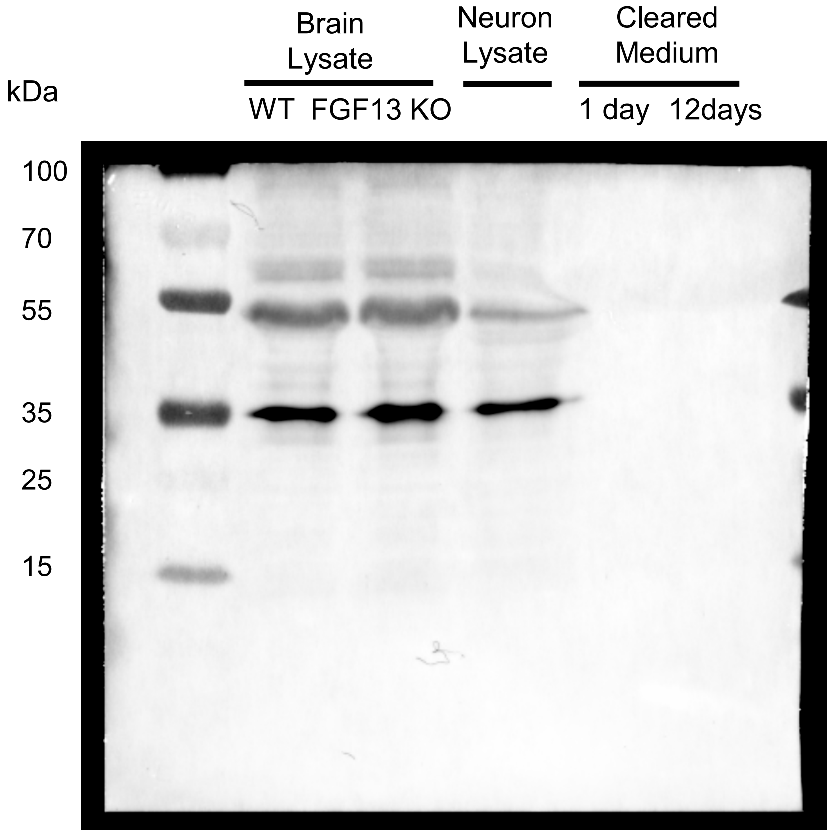

GAPDH
